# Matrix Stiffness Regulates Diffuse Large B-Cell Lymphoma (DLBCL) Spheroid Formation in a Tunable 3D Chitosan Hydrogel Model

**DOI:** 10.3390/gels12080705

**Published:** 2026-08-08

**Authors:** Gaia Corallo, Maria Carmela Vegliante, Susanna Anita Pappagallo, Antonella Stanzione, Francesca Scalera, Giuseppe Gigli, Domenico Pastore, Sabino Ciavarella, Francesca Gervaso, Alessandro Polini

**Affiliations:** 1 Dipartimento di Matematica e Fisica E. de Giorgi, Università del Salento, c/o Campus Ecotekne, 73100 Lecce, Italy; gaia.corallo@unisalento.it; 2 Institute of Nanotechnology, National Research Council (CNR-NANOTEC), c/o Campus Ecotekne, Via Monteroni, 73100 Lecce, Italyfrancesca.scalera@cnr.it (F.S.);; 3 Tecnomed Puglia—Technopole for Precision Medicine (Biotech Lecce Hub), c/o Campus Ecotekne, Via Monteroni, 73100 Lecce, Italy; 4 Hematology and Cell Therapy Unit, IRCCS-Istituto Tumori ‘Giovanni Paolo II’, 70124 Bari, Italy; m.vegliante@oncologico.bari.it (M.C.V.); s.ciavarella@oncologico.bari.it (S.C.); 5 Dipartimento di Medicina Sperimentale, Università del Salento, c/o Campus Ecotekne, 73100 Lecce, Italy

**Keywords:** hydrogels, lymphoma, stiffness, spheroids

## Abstract

Diffuse large B-cell lymphoma (DLBCL) represents the most frequent subtype of non-Hodgkin lymphoma; however, conventional 2D cultures and murine models fail to fully recapitulate the complexity of the human tumor microenvironment (TME). To address these limitations, we developed and characterized three chitosan-based hydrogels with distinct physicochemical and mechanical properties as tunable three-dimensional (3D) platforms for DLBCL culture. U2932 lymphoma cells were encapsulated either alone or in co-culture with WPMY-1 stromal cells to investigate the effects of matrix stiffness and stromal support on lymphoma growth. Furthermore, co-culture with stromal cells promoted rapid spheroid formation and generated larger cellular aggregates than mono-cultures, with the most pronounced effect observed in the 2% chitosan hydrogel. Live/dead assays demonstrated high cell viability within the selected culture condition. Overall, the 2% chitosan formulation provided the most suitable microenvironment for supporting DLBCL growth and organization, highlighting its potential as a physiologically relevant 3D in vitro model for investigating lymphoma biology and evaluating novel therapeutic strategies.

## 1. Introduction

Diffuse large B-cell lymphoma (DLBCL) represents the most prevalently diagnosed lymphoid neoplasm in the adult population, representing approximately 30–40% of B-cell non-Hodgkin lymphomas [1,2,3,4]. It is an aggressive and highly heterogeneous disease characterized by the proliferation of large malignant B cells and a diverse array of genomic aberrations, ranging from point mutations to copy number alterations and structural chromosomal rearrangements [5,6]. Based on gene expression profiling, DLBCL is classified into two major molecular subtypes, germinal center B-cell-like (GCB) and non-GCB, with the latter encompassing the activated B-cell-like (ABC) subtype [7,8]. Although immunochemotherapy with R-CHOP remains the standard first-line treatment, approximately one-third of patients develop refractory or relapsed disease, underscoring the need for improved molecular stratification and more predictive preclinical models to support the development of personalized therapeutic strategies [1,3,9,10].

Although molecular studies have greatly expanded our understanding of DLBCL biology, the lack of physiologically relevant in vitro platforms still limits the investigation of microenvironment-dependent mechanisms and treatment responses [1,11,12]. The DLBCL tumor microenvironment (TME) includes immune and stromal cells, soluble mediators, extracellular matrix (ECM) components, and physical cues such as stiffness, porosity, spatial confinement, and oxygen or nutrient gradients [13,14,15,16]. Among these, ECM mechanics are increasingly recognized as functional regulators of B-cell behavior through mechanotransduction, affecting gene expression, cytoskeletal organization, survival, and drug response [17,18]. In lymphoid malignancies, mechanical alterations are mainly driven by tumor cell accumulation and the disruption of the native stromal and reticular architecture, generating local changes in ECM organization and stiffness within the otherwise soft lymph node microenvironment [19,20,21]. Consistent with this concept, recent work by Apoorva et al. has provided compelling evidence that the mechanical properties of the lymphoid microenvironment actively regulate malignant B-cell function by modulating B-cell receptor (BCR) signaling and integrin activation, thereby influencing cell survival and therapeutic responses [22]. These observations further strengthen the rationale for developing mechanically tunable hydrogel platforms to investigate the role of matrix stiffness in DLBCL biology.

Most current in vitro models are two-dimensional (2D) and fail to reproduce the spatial organization, biophysical constraints, and cell–matrix interactions observed in vivo [16,20,21,23]. Advanced 3D models are therefore needed to better recapitulate lymphoma architecture and TME-dependent signaling while reducing experimental variability [13,24,25]. In this context, hydrogels provide a tunable scaffold in which mechanical and physicochemical properties, including stiffness and hydration, can be controlled to support more physiologically relevant cellular phenotypes [26,27]. Compared with conventional 2D cultures, 3D lymphoma models more closely reproduce tumor growth dynamics, metabolic activity, signaling pathways, and drug responses and may sustain lymphoid-specific processes that are otherwise poorly maintained ex vivo [13,24,28,29,30].

Several hydrogel-based platforms have been proposed to model lymphoma in vitro. Alginate-based hydrogels have been successfully employed to generate multicellular lymphoma spheroids and evaluate therapeutic responses, although they generally require the incorporation of adhesive peptides or additional ECM components to overcome the intrinsic bioinert nature of alginate [16,24]. Similarly, gelatin- and agarose-based platforms have supported lymphoma–stromal interactions and spheroid formation but have primarily served as structural scaffolds rather than as systems specifically designed to investigate the contribution of matrix mechanics [13,31]. Despite these advances, there remains a critical need for straightforward, reproducible, and mechanically tunable hydrogel systems that enable the systematic investigation of the role of extracellular matrix mechanics in lymphoma biology.

Among natural polymers, chitosan is particularly attractive for 3D cell culture applications because of its biocompatibility, biodegradability, structural similarity to glycosaminoglycans, and ability to form thermosensitive hydrogels. Hydrogels composed of chitosan exhibit a temperature-induced sol–gel transition upon reaching body temperature, allowing for homogeneous cell encapsulation under mild conditions, while their mechanical properties can be tuned by varying polymer concentration [32,33,34,35]. These features make chitosan a suitable platform for investigating stiffness-dependent cellular responses in lymphoma.

Beyond the matrix itself, 3D lymphoma models can be further improved by incorporating spheroid organization and stromal components. This is particularly relevant for DLBCL, in which non-malignant TME cells contribute to biological and clinical heterogeneity and actively regulate tumor behavior through biochemical, mechanical, and paracrine interactions [9,14,15,30,31,36,37]. Combining tunable 3D hydrogels with stromal co-culture may therefore provide a more informative system to model lymphoma–microenvironment interactions.

Here, we engineered a thermally responsive 3D chitosan hydrogel system to model DLBCL growth within mechanically tunable microenvironments. By testing three distinct chitosan concentrations (1.2%, 2%, and 3%), we investigated how variations in matrix mechanics influence the viability, organization, and spheroid formation of encapsulated U2932 cells. To incorporate a stromal component of the lymphoma niche, cells were cultured either alone or in co-culture with WPMY-1 stromal cells. The 2% chitosan hydrogel emerged as the optimal condition, providing a physicochemical environment that promoted U2932 self-aggregation into stable spheroids—an effect further enhanced by stromal co-culture. By integrating tunable mechanical properties with key cellular components of the tumor niche, this biomimetic platform enables a more faithful reconstruction of the DLBCL microenvironment and underscores the critical role of matrix stiffness in regulating lymphoma cell behavior, offering a versatile and physiologically relevant tool for investigating lymphoma–microenvironment interactions.

## 2. Results and Discussion

**Figure 1:**
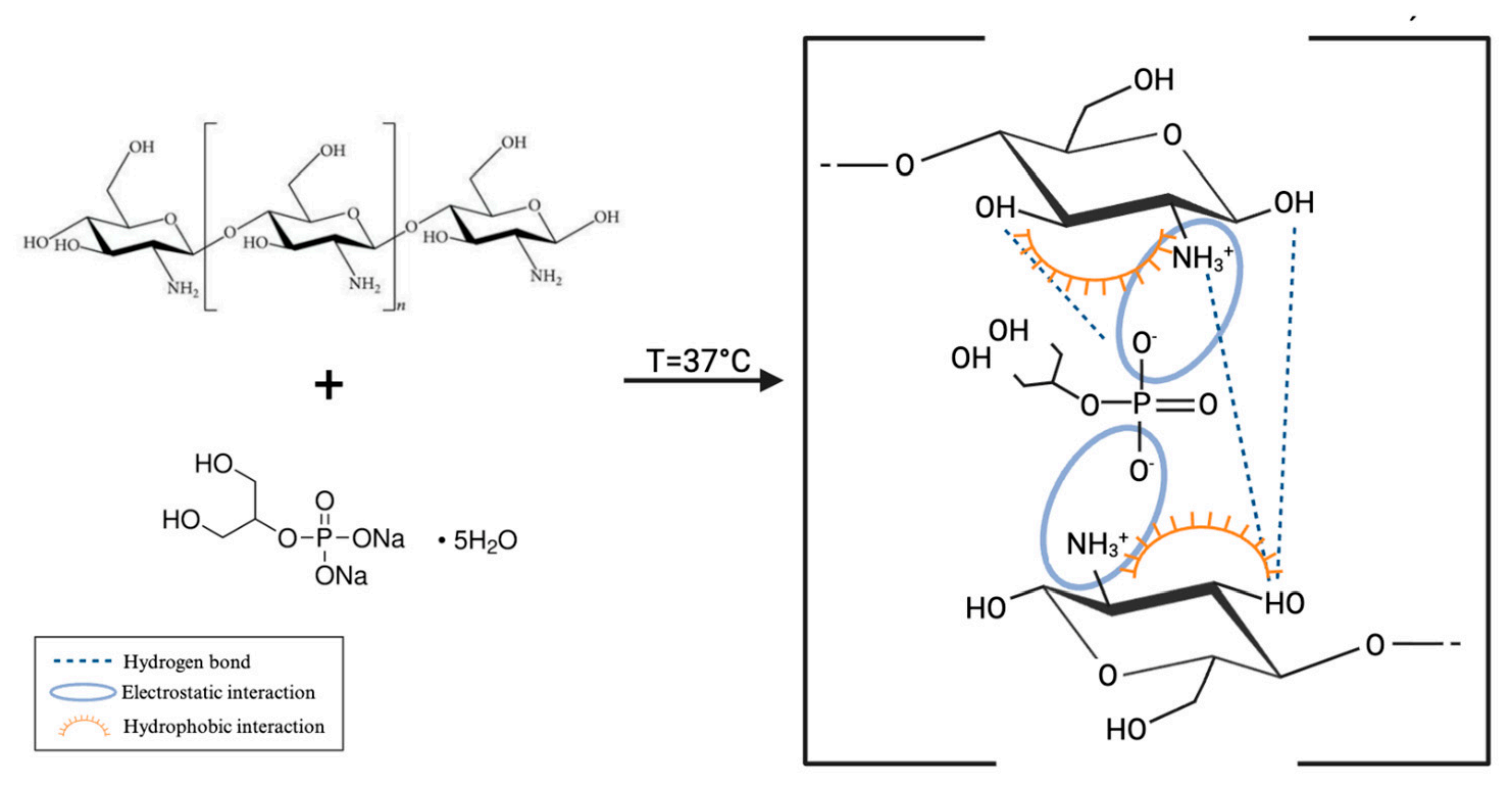
A schematic representation of the molecular interactions involved in the formation of the Ch/β-glycerophosphate thermoresponsive hydrogel. Electrostatic interactions between protonated chitosan amino groups (–NH_3_^+^) and phosphate groups of β-glycerophosphate, together with hydrogen bonding and hydrophobic associations between chitosan chains, contribute to the formation of a physically crosslinked polymeric network.

Chitosan-based (Ch) hydrogels at different concentrations were generated through the thermoresponsive interaction between Ch and β-glycerophosphate (β-GP), which promotes the formation of a 3D physical network suitable for cell encapsulation. As schematically represented in Figure 1, hydrogel formation is mediated by multiple non-covalent interactions, including electrostatic associations between protonated amino groups of chitosan (–NH_3_^+^) and phosphate groups of β-GP, hydrogen bonding, and hydrophobic interactions among chitosan chains. Upon increasing temperature to physiological conditions (37 °C), reduced polymer hydration and decreased electrostatic repulsion facilitate Ch chain association, resulting in a sol–gel transition and the formation of a stable hydrated matrix capable of entrapping U2932 and WPMY−1 cells [34,38,39].

Ch hydrogel formulations with different polymer concentrations were prepared and characterized from physicochemical and biological perspectives.

### 2.1. FTIR Analysis Confirms the Physical Interactions Within Ch/β-GP Hydrogels

**Figure 2:**
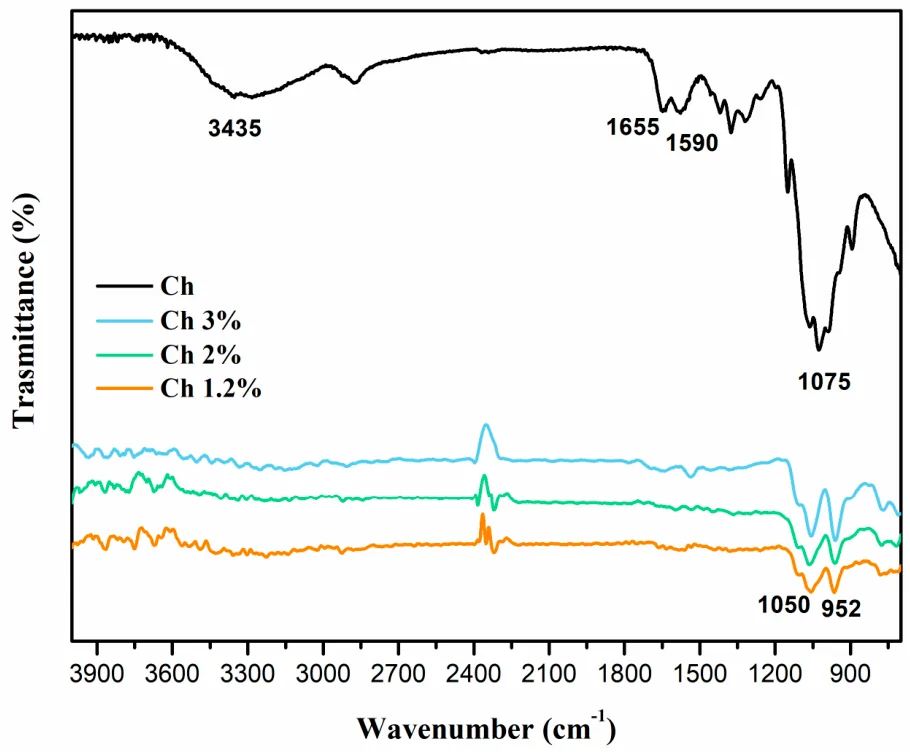
The FTIR spectra of pure chitosan and dried Ch/β-GP films containing 1.2%, 2%, and 3% chitosan, recorded over the 4000–400 cm^−1^ range.

The FTIR spectra of pure chitosan and the 1.2%, 2%, and 3% Ch/β-GP formulations are displayed in Figure 2. Pure chitosan displayed the characteristic broad O–H/N–H stretching band centered at approximately 3435 cm^−1^, together with bands at approximately 1655 and 1590 cm^−1^, attributed to amide I and primary amino group vibrations, respectively. A strong absorption band was also observed around 1075 cm^−1^ and assigned to the C–O and C–O–C stretching modes within the saccharide ring structure.

Compared with pure chitosan, the spectra of the Ch/β-GP formulations showed marked changes in the O–H/N–H stretching region and in the 1200–900 cm^−1^ range. In particular, the broad band centered at approximately 3435 cm^−1^ and the primary amine-associated band at approximately 1590 cm^−1^ were no longer clearly resolved in the hydrogel films. These variations may be attributed to the partial deprotonation and charge screening of the protonated chitosan amino groups, together with hydrogen bonding and electrostatic interactions between the remaining –NH_3_^+^ groups and the phosphate groups of β-GP.

The Ch/β-GP formulations also showed broad contributions around 1050 and 952 cm^−1^. This spectral range contains overlapping C–O and C–O–C vibrations of chitosan and phosphate-associated vibrations of β-GP. In analogous Ch/β-GP systems, the asymmetric and symmetric stretching vibrations of phosphate groups have been reported at approximately 1080 and 968 cm^−1^, respectively [40].

The observed changes support β-GP incorporation and the establishment of intermolecular physical interactions within the formulations. No new absorption bands attributable to covalent bond formation were detected, consistently with the physical nature of the Ch/β-GP network.

### 2.2. Chitosan Hydrogels Display Injectability and Thermoresponsive Gelation Under Physiological Conditions

The pH levels of the individual GA and chitosan solutions were recorded both prior to and following their combination. Although the starting Ch solutions were acidic (pH = 3 for Ch 1.2% and 3.33%; pH = 5 for Ch 5%), all Ch systems reached a near-neutral value (pH ~ 7) immediately after mixing, compatible with cell encapsulation and culture conditions (Table 1).

**Table 1:** Physicochemical characteristics of Ch hydrogels at different concentrations (3%, 2%, and 1.2%).

Final Concentration of Ch Hydrogels	pH	Injectability	Thermal Polymerization/ Thermoresponsiveness at 37 °C	Flow Speed (Inversion Tube Test)	Macroscopic Appearance
3%	6.9	Yes	35–45 min	Very slow	Homogeneous, cloudy/opaque, highly viscous
2%	7.4	Yes	45’- 1 h	Slow	Homogeneous, cloudy/opaque, viscous
1.2%	7.7	Yes	1 h	Very fast	Homogeneous, clear, liquid

**Figure 3:**
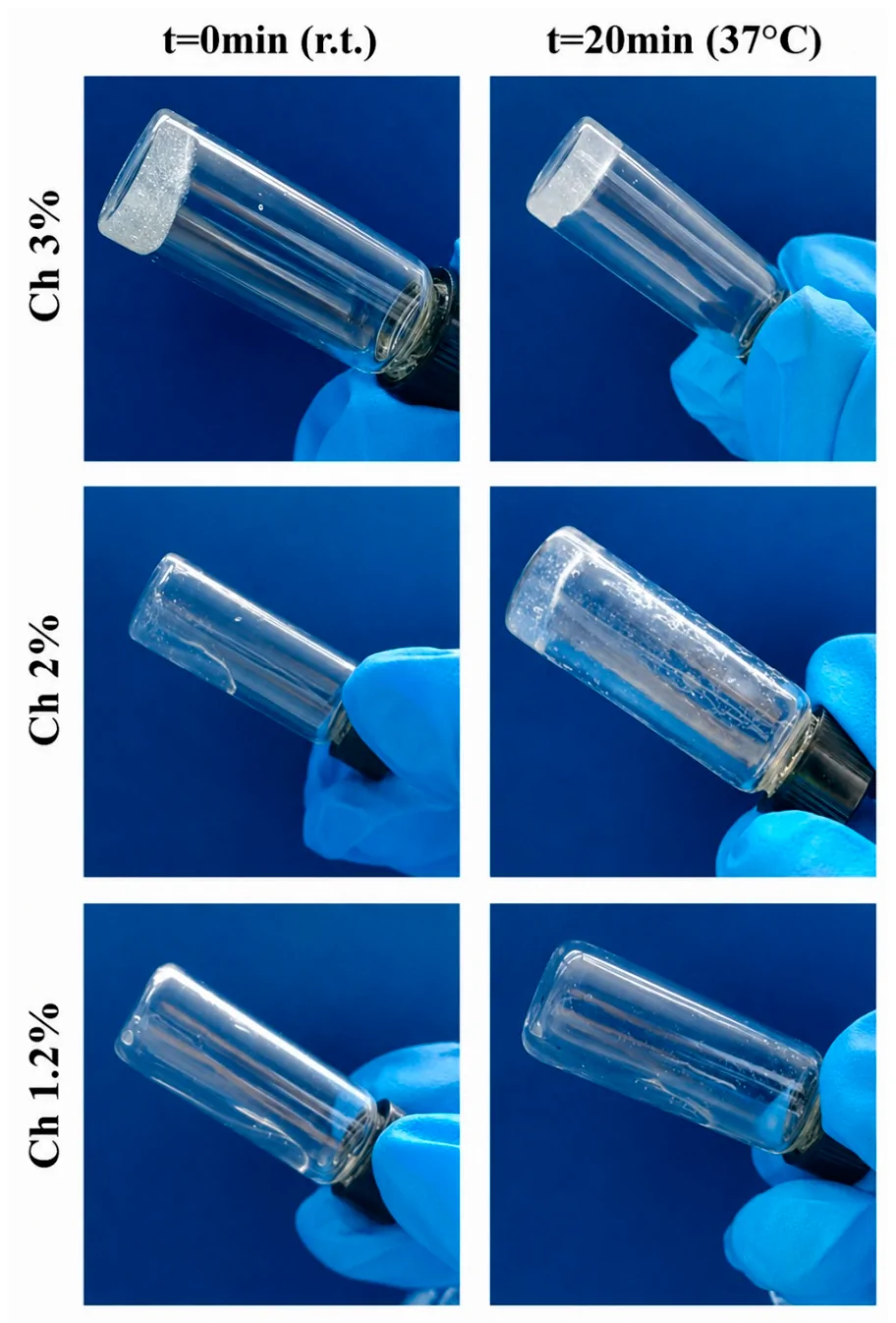
Inversion tube test: Images of the three hydrogel formulations taken immediately after mixing at r.t. and after 20 min of incubation at 37 °C.

An inversion tube test was performed to assess hydrogel thermoresponsiveness and compare gelation behavior at different concentrations (Figure 3). All hydrogels flowed along the vial walls promptly after mixing at r.t. However, they stopped flowing after 20 min at 37 °C, indicating a successful sol–gel transition. The formulation with the lowest polymer concentration (1.2% Ch) flowed faster than the higher-concentration formulations, which were characterized by higher viscosity.

The injectability of the three Ch formulations was assessed and confirmed by extruding the hydrogels through a 23 G needle. All three formulations appeared homogeneous. Visually, the 1.2% Ch hydrogel was clearer and less viscous than the two formulations with higher Ch concentrations.

### 2.3. Chitosan Concentration Controls Hydrogel Stiffness Within a Lymphoid Tissue-Relevant Range

Emerging evidence highlights biomechanical forces and 3D architecture as major contributors to lymphoma development and therapeutic resistance, with mechanical, structural, and topographical ECM alterations correlating with histotype and tumor grade [18]. In this context, Ch represents an attractive scaffold for cell encapsulation: its glycosaminoglycan-like structure provides a biomimetic microenvironment resembling native ECM components [33,38,41], while its biocompatibility, mild gelation conditions, and tunable mechanical properties enable the recapitulation of physiologically relevant stiffness ranges [42,43]—a parameter identified as a key regulator of DLBCL spheroid formation [44].

**Figure 4:**
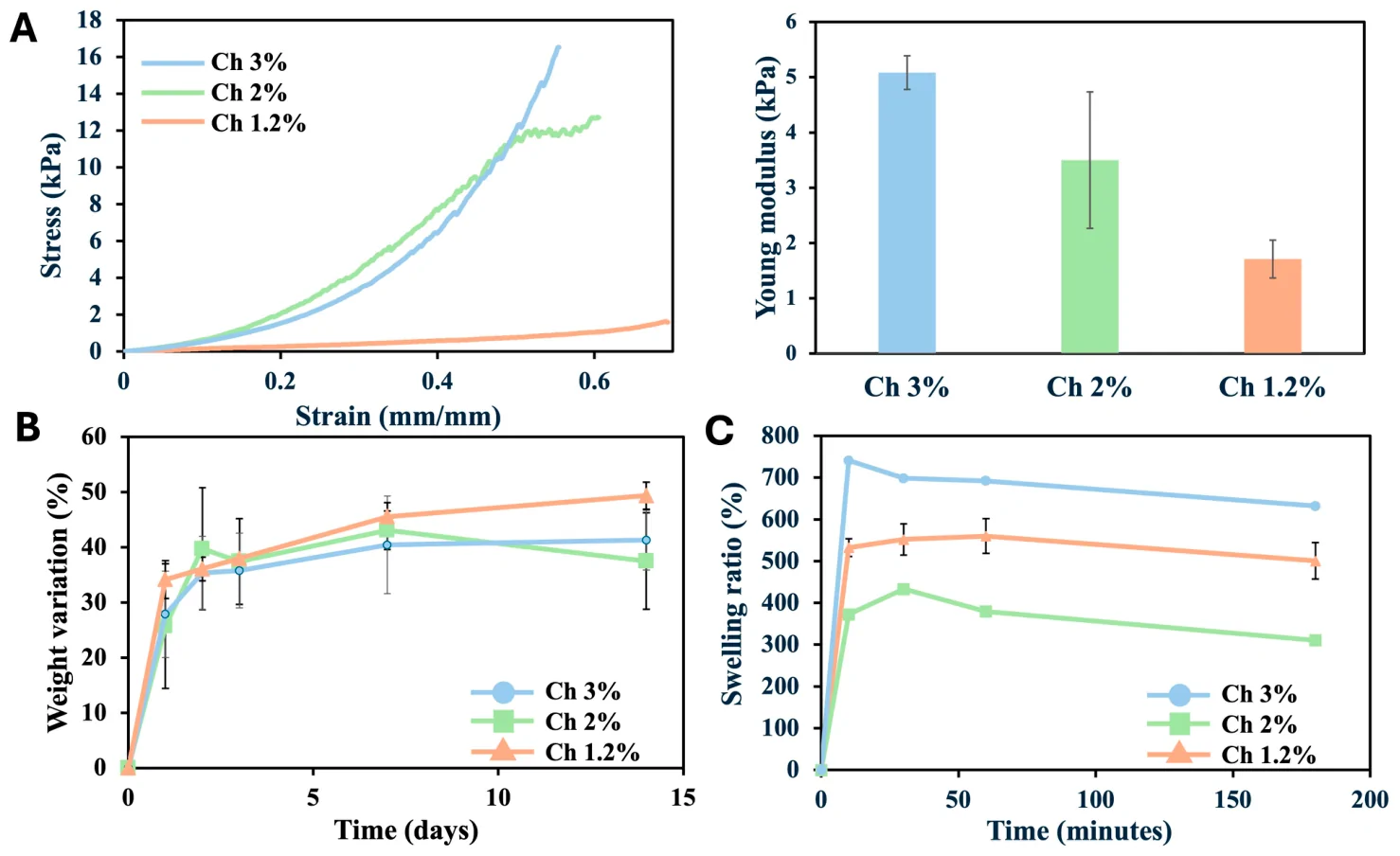
(**A**) The stress–strain compression test performed on 3% Ch, 2% Ch, and 1.2% Ch hydrogels. The secant modulus was calculated as the slope of a line connecting zero strain to a point at a specified deformation from the stress–strain curves. Right panel: The Young’s modulus of the three hydrogel formulations. (**B**) Stability test: The weight loss of Ch hydrogels at different concentrations (3%, 2%, and 1.2%) immersed in PBS at 37 °C. (**C**) The swelling behavior of Ch hydrogels at different concentrations (3%, 2%, and 1.2%).

Compression testing revealed a direct correlation between Ch polymer concentration and hydrogel stiffness (Figure 4A). The Young’s modulus calculated during unconfined compression at low strain values (0–5%) was approximately 1.7 kPa for 1.2% Ch, 3.5 kPa for 2% Ch, and 5 kPa for 3% Ch.

### 2.4. Physicochemical Characterization Reveals Distinct Properties of Chitosan Hydrogel Formulations

The degradation profile of the 3%, 2%, and 1.2% chitosan (Ch) hydrogels was evaluated by measuring sample weight variation during incubation in PBS for 14 days (Figure 4B). After an initial increase in weight associated with hydration, all formulations exhibited minimal changes over time, demonstrating good mass retention. At day 14, the 3% and 2% Ch hydrogels displayed weight variations of approximately 40%, while the 1.2% formulation reached approximately 50%. These results indicate that all the hydrogels remained stable throughout the experimental period and were compatible with the duration required for cell culture studies.

To evaluate swelling behavior, the mass of the lyophilized samples was recorded prior to and following hydration. All hydrogel formulations exhibited rapid PBS uptake, with most absorption occurring within the first 10 min, followed by negligible release over the next 3 h (Figure 4C). The 3% Ch hydrogel exhibited the highest swelling capacity, whereas the 2% formulation showed the lowest. The 1.2% Ch hydrogel displayed intermediate swelling behavior. Overall, swelling did not show a linear dependence on Ch concentration.

This non-linear swelling behavior indicates that water uptake is not governed by Ch content alone but rather by the balance between hydrophilicility-driven osmotic forces, the elastic constraints imposed by the polymeric network, and the accessibility of the resulting pore architecture.

At higher Ch concentration (3%), the abundance of hydrophilic functional groups likely enhances water binding, and the resulting osmotic pressure overcomes the increased crosslink density, leading to elevated swelling [38]. In contrast, the 2% formulation appears to represent a regime in which polymer chain density and β-GP-mediated crosslinking imposes sufficient elastic constraints to limit network expansion, while the number of hydrophilic sites is not yet high enough to compensate. As a result, both water uptake and volumetric expansion are minimized. At the lowest concentration (1.2%), the reduced network density yields a more compliant structure with higher chain mobility, allowing for greater relative expansion despite lower intrinsic water-binding capacity [45].

**Figure 5:**
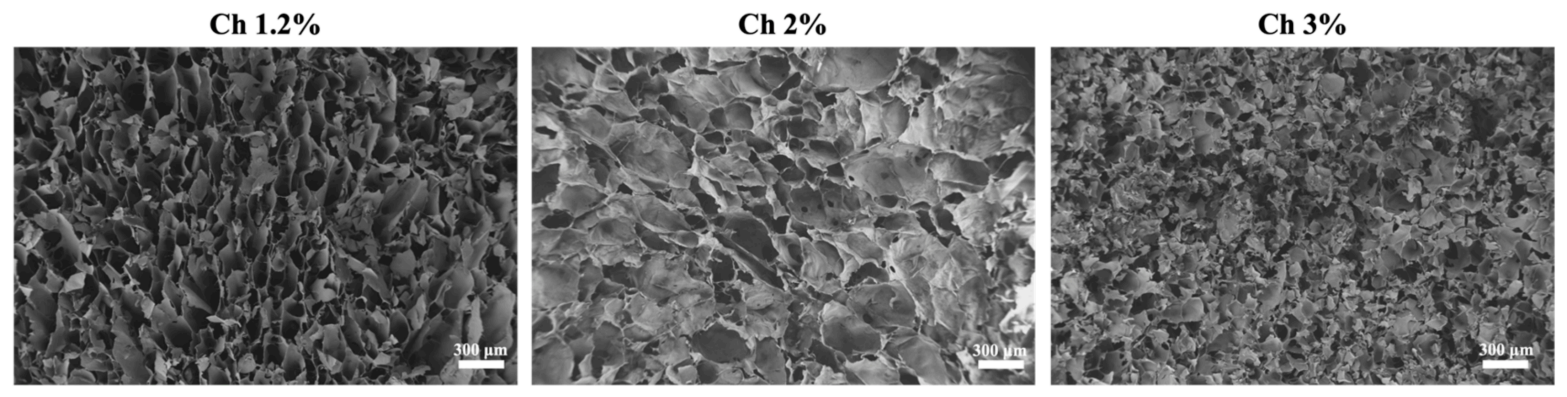
Representative SEM micrographs of freeze-dried cross-sections of the 1.2%, 2%, and 3% Ch hydrogels acquired at comparable magnifications. Scale bars are shown in each image.

The SEM observations support this interpretation (Figure 5).

The 1.2% and 3% Ch hydrogels exhibited an open, sponge-like morphology with numerous interconnected voids, whereas the 2% Ch formulation displayed a more compact, predominantly lamellar organization, with irregular cavities that appeared less interconnected. Although large voids were locally visible in the 2% Ch hydrogel, they did not form the continuous sponge-like architecture observed at 1.2% and 3% Ch. Since swelling was measured on freeze-dried samples, the accessibility and interconnectivity of the dried pore network directly influenced PBS penetration and capillary retention; accordingly, the lower swelling of the 2% Ch hydrogel is most plausibly related to its reduced accessible pore volume and limited pore connectivity, rather than to polymer concentration alone.

These structural differences might arise, at least in part, from concentration-dependent changes in the physical organization of the Ch/β-GP system [46]. Although the final β-GP concentration was maintained constant across formulations, increasing the Ch concentration progressively increases the number of protonated amine groups available for electrostatic screening, thereby altering the effective β-GP-to-Ch ratio rather than uniformly increasing crosslink density with polymer content. β-GP is known to reduce electrostatic repulsion among protonated chitosan chains through charge screening and partial neutralization, facilitating polymer–polymer association during gelation (Figure 1). At 2% Ch, this ratio may fall within a favorable compositional window in which the available β-GP is sufficient to adequately neutralize the Ch amine groups, while chain mobility remains high enough to permit relatively homogeneous chain overlap and physical association. This would be consistent with the compact, lamellar structure observed by SEM, in which well-distributed crosslinking imposes elastic constraints that limit network expansion and, consequently, swelling.

At 3% Ch, the same fixed β-GP concentration must screen a substantially larger population of Ch amine groups, and we hypothesize that the available β-GP becomes stoichiometrically insufficient to achieve complete, spatially homogeneous neutralization at this concentration. Combined with the higher viscosity and more extensive chain entanglement characteristic of concentrated chitosan solutions, this incomplete and uneven charge screening may limit molecular diffusion and homogeneous rearrangement during gelation, preserving local polymer-poor, water-rich domains that collapse into an open, sponge-like structure upon freezing and lyophilization. Under this interpretation, the elevated swelling observed at 3% Ch would reflect an effectively looser and more heterogeneous network. The abundant hydrophilic groups on chitosan likely still contribute to water binding within this loosely organized network, but the magnitude of swelling appears to be dominated by the accessibility and interconnectivity of the pore architecture rather than by hydrophilicity alone.

At 1.2% Ch, a distinct mechanism likely underlies the comparably open morphology and elevated swelling. Here, the low absolute Ch content limits overall chain density and network connectivity, even though the β-GP-to-chitosan ratio is proportionally more favorable at this concentration than at 3% Ch. The resulting structure is therefore open but mechanically weak, consistent with the SEM observations, reflecting insufficient chain density rather than crosslinker imbalance.

Taken together, these results suggest that swelling behavior in this Ch/β-GP hydrogel system is governed by the combined effects of hydrophilicity-driven water uptake and the effective, spatially homogeneous crosslink density set by the β-GP-to-Ch ratio, which appears to be optimal near 2% Ch. Deviations from this optimum—arising from insufficient absolute polymer density at 1.2% Ch or from a relative crosslinker deficiency at 3% Ch—result in more open, interconnected network architectures and correspondingly higher swelling. The qualitative agreement between the swelling data and the SEM-derived microarchitecture supports this interpretation, although we acknowledge that the direct quantification of the β-GP:Ch molar ratio and controlled freeze-drying studies would be needed to confirm the proposed mechanism and to disentangle crosslinking effects from freeze-drying artifacts.

### 2.5. Thermosensitive Chitosan Hydrogels Acquire Solid-like Viscoelastic Properties at 37 °C

**Figure 6:**
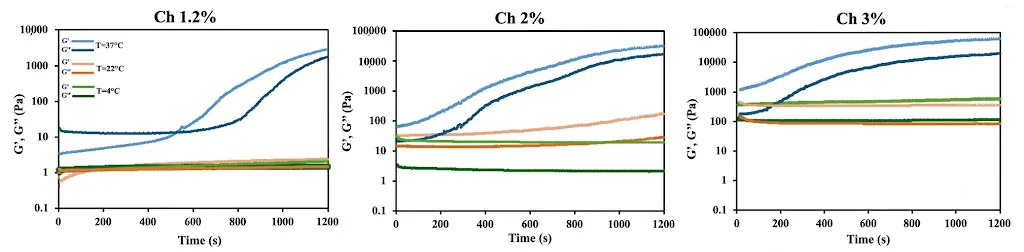
Time sweep measurement showing the kinetics of elastic (G′) and viscous (G″) moduli at 1 Hz and 10% strain for Ch hydrogels at three concentrations (1.2%, 2%, and 3% *w*/*v*) and three temperatures: 4 °C (green), 22 °C (orange/peach), and 37 °C (blue). Lighter shades represent the storage modulus (G′), while darker shades represent the loss modulus (G″). The time sweep was conducted over 20 min (1200 s).

Dynamic rheometry was performed to monitor the viscoelastic evolution of the hydrogels. Time sweep tests were conducted at constant frequency for 20 min at three temperatures (4 °C, 22 °C, and 37 °C), as reported in Figure 6. For the 1.2% Ch formulation at 37 °C, a crossover point between the storage modulus (G′) and loss modulus (G″) was observed. Initially, the sample exhibited a predominantly viscous response (G″ > G′), but over time G′ increased until it surpassed G″, marking the gelation point. After this crossover, G′ remained higher than G″, indicating a transition to a solid-like state. In contrast, the 2% and 3% Ch formulations displayed no intersection between G′ and G″. In these samples, G′ remained consistently and markedly higher than G″ throughout the time sweep. Regarding temperature dependence, G′ and G″ values for all formulations remained relatively stable at 4 °C and 22 °C, whereas at 37 °C both moduli increased over time until reaching a plateau. Quantitatively, the 1.2% hydrogel exhibited lower overall moduli than the 2% and 3% formulations, which reached higher stiffness values.

### 2.6. Matrix Stiffness and Cell Density Regulate U2932 Aggregation in 3D Culture

To identify the optimal conditions for DLBCL modeling, U2932 cells were cultured within the three hydrogel formulations in mono-culture or in co-culture with WPMY-1 cells and monitored for 14 days.

**Figure 7:**
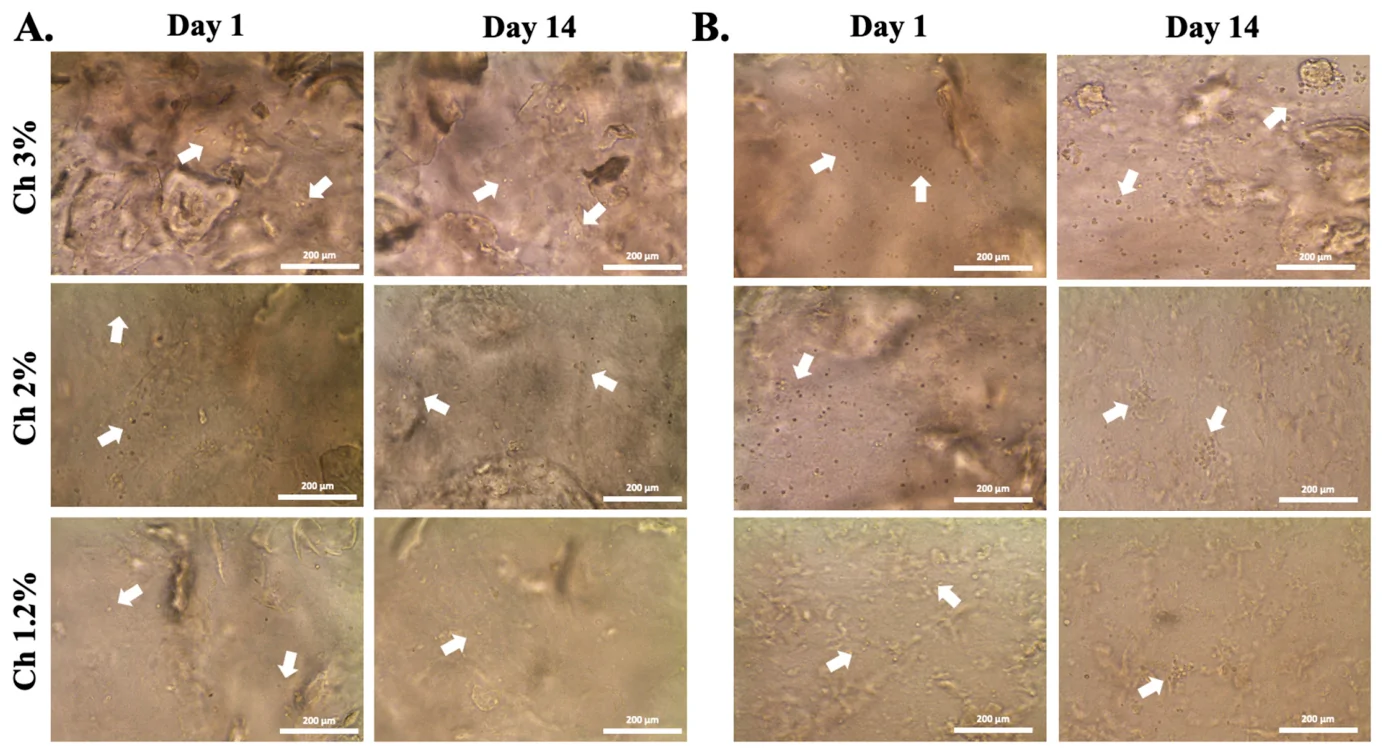
Bright-field images of U2932 cells encapsulated within three hydrogel formulations (3% Ch, 2% Ch, and 1.2% Ch) at a concentration of 4 × 10^6^ cells/mL of hydrogel. (**A**) U2932 cells encapsulated in mono-culture conditions. (**B**) U2932 cells encapsulated in co-culture conditions with 10% WPMY-1 cells. White arrows indicate representative encapsulated cells. Scale bars: 200 μm.

As reported in Figure 7, cells encapsulated at 4 × 10^5^ cells/mL displayed a slight increase in cell density over 14 days of culture in both mono- and co-culture conditions. However, cells remained homogeneously distributed within the matrices across all concentrations and did not form tumor-like spheroids.

**Figure 8:**
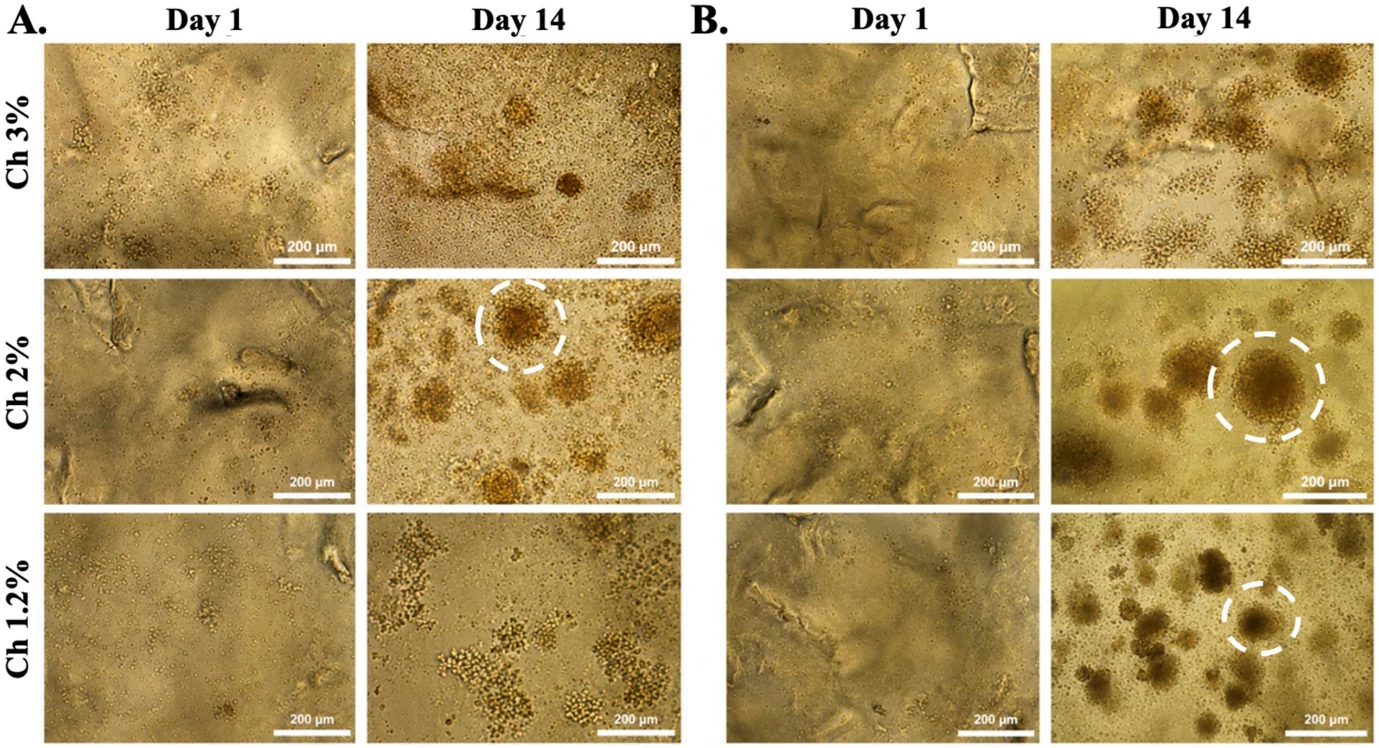
Bright-field images of U2932 cells encapsulated within three hydrogel formulations (3% Ch, 2% Ch, and 1.2% Ch) at a concentration of 4 × 10^6^ cells/mL of hydrogel. (**A**) U2932 cells encapsulated in mono-culture conditions. (**B**) U2932 cells encapsulated in co-culture conditions with 10% WPMY-1 cells. Dashed circles highlight representative cell aggregates. Scale bars: 200 μm.

In contrast to the lower-density condition, the cell concentration of 4 × 10^6^ cells/mL induced aggregation into spheroids in most formulations (Figure 8). In the highest-concentration hydrogel (3% Ch), cells did not aggregate in either mono- or co-culture conditions, although high cell proliferation was observed. In the lowest-concentration hydrogel (1.2% Ch), spheroid formation occurred in both mono- and co-culture conditions. In particular, the co-culture group displayed faster aggregation and higher U2932 cell proliferation from the first days of culture. However, spheroids in this matrix remained small throughout the 14-day culture period.

The 2% Ch formulation supported the most robust aggregation, with cells forming large spheroids in both mono- and co-culture conditions. Notably, the co-culture of U2932 and WPMY-1 cells led to the formation of significantly larger spheroids (200–250 µm) in a shorter time and with a higher proliferation rate than mono-culture.

This suggests that DLBCL organization in 3D depends on the combined contribution of matrix mechanics and local cell–cell contact [47,48,49,50]. This observation is biologically relevant, since lymphomatous lymph nodes are characterized by dense tumor infiltration and the progressive disruption of the native architecture; therefore, seeding density should be considered not only a technical variable but also a determinant of spatial organization, mechanical stress, and paracrine signaling. A further important observation was that stromal co-culture enhanced spheroid formation. The addition of 10% WPMY-1 stromal cells accelerated aggregation and increased spheroid size, particularly within the 2% Ch hydrogel. WPMY-1 cells were selected as a reproducible ACTA2/α-SMA-positive myofibroblast-like stromal model [51]. However, because they are prostate-derived, they should not be considered a substitute for lymph node-derived FRCs and cannot fully reproduce their specialized functions within the lymphoma microenvironment. Although WPMY-1 cells represent a simplified stromal surrogate rather than an autologous lymph node-derived population, these findings indicate that even a minimal stromal component can substantially influence DLBCL organization in 3D. Indeed, beyond matrix mechanics, the DLBCL microenvironment is shaped by dynamic interactions between tumor cells and stromal, immune, and vascular components [52]. Among these, cancer-associated fibroblasts (CAFs) actively remodel both the biochemical and mechanical properties of the tumor microenvironment, regulating lymphoma progression and therapeutic response [53,54]. In DLBCL, stromal signatures such as Stromal-1 have been associated with lymph node remodeling and coordinated interactions among tumor, stromal, and immune cells [55], while lymphoma cells can reprogram stromal fibroblasts toward immunosuppressive phenotypes through reciprocal pro-tumoral signaling pathways [37,56]. Collectively, these observations support the biological relevance of incorporating stromal components into 3D lymphoma models.

### 2.7. Chitosan-Based 3D Matrices Support High Cell Viability over Time

**Figure 9:**
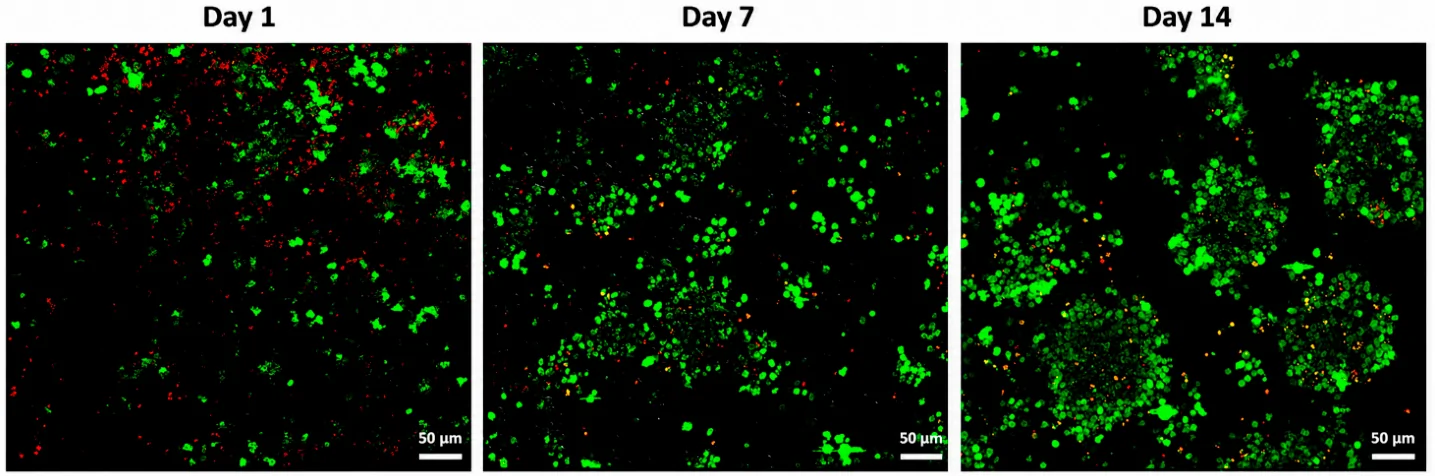
Live/dead (green/red) images of U2932/WPMY-1 cells encapsulated within 2% Ch hydrogel at three time points (day 1, day 7, and day 14). Scale bars: 50 μm.

Based on the physicochemical characterization, the 2% Ch hydrogel was selected for subsequent viability assessment. A live/dead assay was performed 1, 7, and 14 days after the encapsulation of U2932/WPMY-1 cells (Figure 9). Cells remained viable throughout the 14-day culture period. Only rare dead cells, indicated by red nuclei, were detected, confirming the suitability of the 2% Ch hydrogel as a matrix for long-term cell encapsulation.

The encapsulation strategy adopted in this study also contributes to the suitability of the proposed platform to maintain viability. Cells were suspended in the liquid Ch/β-glycerophosphate precursor before gelation, allowing for homogeneous distribution throughout the matrix. Upon incubation at 37 °C, β-glycerophosphate induced the thermosensitive gelation of Ch, generating a 3D polymer network that physically retained the cells within the hydrogel. Besides acting as a GA, β-glycerophosphate neutralizes the acidic Ch solution, providing physiological pH conditions compatible with cell viability while avoiding the use of potentially cytotoxic chemical crosslinkers [34,39].

## 3. Conclusions

The thermosensitive Ch/β-glycerophosphate hydrogel developed in this study provides a simple, injectable, and reproducible platform for 3D DLBCL modeling with tunable mechanical properties. By modulating Ch concentration, it was possible to systematically investigate the influence of matrix stiffness on lymphoma spheroid organization within a chemically defined and cell-compatible environment while retaining the flexibility to incorporate stromal components. Among the investigated formulations, the 2% Ch hydrogel exhibited the most suitable physicochemical and mechanical characteristics, supporting the formation of viable, stable, and stromal-enhanced DLBCL spheroids. These findings demonstrate that matrix mechanics, together with stromal interactions, play a critical role in regulating lymphoma organization and highlight the importance of biomaterial-based platforms for investigating lymphoma mechanobiology. Although the current model does not yet fully recapitulate the cellular and biochemical complexity of the lymph node microenvironment, its simplicity, reproducibility, and tunable properties provide a strong foundation for future refinement and expansion. The platform can be readily adapted through the incorporation of additional ECM components, immune and endothelial cell populations, and other relevant microenvironmental cues. Furthermore, its modular design enables its application beyond the current cell lines, supporting the investigation of different lymphoma subtypes and other disease-relevant cellular models, with future extension toward patient-derived cells and personalized disease modeling approaches, including integration with organ-on-chip technologies. Overall, this platform represents a promising complementary preclinical tool for investigating lymphoma–microenvironment interactions and for facilitating the development and evaluation of novel therapeutic strategies within physiologically relevant three-dimensional culture systems.

## 4. Materials and Methods

### 4.1. Chitosan-Based Hydrogel Preparation

Low-molecular-weight Ch (#448869, Sigma-Aldrich, Milan, Italy) was used to prepare three polymer solutions at different concentrations (5%, 3.33%, and 2%) by dissolving Ch powder in 0.1 M HCl (Sigma-Aldrich). The solutions were kept under continuous agitation at room temperature (r.t.) overnight. The gelling agent (GA) solution was prepared by dissolving β-glycerophosphate (β-GP, Sigma-Aldrich) in Milli-Q water to a final concentration of 0.5 M. The final hydrogels were prepared by mixing the Ch solution with the GA solution at a 3:2 volume ratio under cold conditions, obtaining final Ch formulations of 3%, 2%, and 1.2%.

### 4.2. Fourier Transform Infrared Spectroscopy

Fourier transform infrared (FTIR) spectroscopy was performed to investigate the chemical features of chitosan and the molecular interactions occurring in the Ch/β-GP formulations. Spectra were acquired using an FT/IR-6300-type JASCO spectrophotometer (Easton, MD, USA) fitted with an attenuated total reflectance (ATR) module. Pure chitosan and freeze-dried films obtained from the 1.2%, 2%, and 3% Ch/β-GP formulations were placed directly onto the ATR crystal and analyzed under the same experimental conditions. Spectra were recorded in the range of 4000–400 cm^−1^ at a resolution of 4 cm^−1^, averaging 100 scans for each sample. A background spectrum was collected before each measurement.

### 4.3. Hydrogel Physicochemical Characterization

Following synthesis, the pH of the Ch/β-GP solutions was measured immediately after mixing and prior to gelation using a calibrated digital pH meter (pH50, XS Instruments, Carpi, Italy), to verify the biocompatibility of the material. Injectability was evaluated by extruding the hydrogel through a syringe equipped with a 23 G needle. In addition, an inversion tube test was performed to assess the gelation time of the three hydrogel formulations. Gelation behavior was determined by monitoring the presence or absence of flow along the walls of a glass tube inverted at 5 min intervals at 37 °C.

### 4.4. Stability Test

Stability was assessed to evaluate the in vitro mass loss behavior of the Ch hydrogel formulations under non-enzymatic physiological conditions. Hydrogels were placed on a glass slide and immersed in phosphate-buffered saline (PBS). Their stability was monitored by measuring the change in hydrogel weight at predetermined time points (1 and 24 h and 3, 7, and 14 days). The percentage of weight loss (WL) was calculated according to the following equation:

(1) WL (%) = [(W_0_ − W_i_)/W_0_] × 100

where W_0 represents the initial weight of the hydrogel after gel formation at 37 °C, and W_t corresponds to the hydrogel weight measured at each time point.

### 4.5. Swelling Test

The swelling behavior of the Ch hydrogel formulations was evaluated by immersing freeze-dried hydrogels in PBS at 37 °C. The dry weight of each sample (W_{dry}) was recorded prior to hydration, while the hydrated weight (W_{wet}) was measured at predetermined time points after PBS incubation. The swelling ratio (SR) was calculated according to the following equation:

SR (%) = [(Wwet − Wdry)/Wdry] × 100

where W_{dry} represents the initial weight of the freeze-dried hydrogel, and W_{wet} corresponds to the hydrogel weight after hydration at each time point.

### 4.6. Compression Test

The mechanical properties of the hydrogel formulations were characterized through uniaxial compression testing. Samples were analyzed 2 h after gelation at room temperature and subsequent equilibration in PBS. Uniaxial compression tests were conducted on a universal testing machine (ZwickiLine 1 kN, ZwickRoell, Kennesaw, GA, USA) fitted with a 10 N load cell. Hydrogels were compressed up to 75% of their initial height at a constant displacement rate of 2 mm min^−1^. To account for the non-linear mechanical response typically exhibited by hydrogels, the secant Young’s modulus was determined. This parameter was calculated as the slope of the stress–strain curve between zero strain and the selected strain value, and values up to 50% strain were used to characterize the evolution of hydrogel stiffness.

### 4.7. Scanning Electron Microscopy

The porous microstructure of the hydrogels was examined by scanning electron microscopy (SEM) using a Zeiss Sigma 300 VP field-emission scanning electron microscope (FE-SEM; Carl Zeiss AG, Oberkochen, Germany). After thermal gelation, the samples were frozen and freeze-dried under the same conditions used for the swelling test. Cross-sectional surfaces were obtained by cutting the dried samples. Before imaging, the specimens were sputter-coated with a 10 nm thick gold layer using a CCU-010 HV coating unit (Safematic GmbH, Zizers, Switzerland). Images were acquired at an accelerating voltage of 5 kV. Micrographs were collected at comparable magnifications to qualitatively compare pore morphology, pore interconnectivity, and polymer–wall organization among the 1.2%, 2%, and 3% Ch formulations.

### 4.8. Rheological Test

The rheological properties of the hydrogel formulations were assessed using a rheometer (Physica MCR 301, Anton Paar, Graz, Austria). Time sweep tests were performed at a frequency of 1 Hz and 10% strain. Approximately 150 µL of each hydrogel solution was placed onto the stage, and a gap distance of 120 µm was set. Samples were left to equilibrate at the target temperature for a few minutes before testing. Rheological measurements were performed using two different geometries, selected according to the hydrogel viscosity. For highly viscous materials, a 25 mm parallel-plate geometry was used, with measurements conducted at 22 °C and 37 °C. For low-viscosity hydrogels, a cone-plate geometry was employed, with tests carried out at 4 °C. All tests were performed in triplicate.

### 4.9. Cell Culture and Cell Encapsulation Within the Hydrogel

The DLBCL cell line U2932 (ACC 633) was purchased from the DSMZ Human and Animal Cell Lines Bank, whereas the stromal cell line WPMY-1 (CRL-2854) was purchased from ATCC. U2932 cells were cultured in RPMI 1640 medium supplemented with 10% fetal bovine serum (FBS), 1% L-glutamine, and 1% penicillin–streptomycin. WPMY-1 cells were cultured in DMEM (D5671, Sigma-Aldrich) complemented with 10% FBS, 1% glutamine, and 1% penicillin–streptomycin. Both cell lines were maintained at 37 °C in a 5% CO_2_ incubator.

U2932 cells were encapsulated within the different hydrogel formulations either in mono-culture or in co-culture with WPMY-1 cells. For each condition, two U2932 cell concentrations were tested (4 × 10^5^ and 4 × 10^6^ cells/mL of hydrogel). In co-culture conditions, WPMY-1 cells accounted for 10% of the total cell number. Cells were suspended in the Ch/β-glycerophosphate precursor solution before gelation and carefully mixed to ensure homogeneous distribution within the matrix. Drops (50 µL) of cell-laden hydrogel were placed into a 24-well culture plate and incubated at 37 °C to induce the sol–gel transition of the thermosensitive biomaterial. After 15–20 min, depending on hydrogel volume, complete medium was added to each well.

### 4.10. Live and Dead Assay

Cell viability within the hydrogel constructs was assessed using a live/dead staining assay at days 1, 7, and 14 after cell encapsulation. The staining solution was prepared by diluting 1.25 µL of Calcein–AM stock solution (4 mM) and 5 µL of propidium iodide stock solution (20 mM) in 2 mL of pre-warmed PBS. Prior to staining, the culture medium was removed from the 24-well plates and replaced with the freshly prepared working solution. Hydrogel samples were incubated at 37 °C for 1 h under dark conditions to allow for the fluorescent labeling of viable and non-viable cells. Following incubation, the staining solution was discarded, and fluorescence images were acquired using a confocal laser scanning microscope (LSM 700, ZEISS, Jena, Germany).

## Data Availability

The data presented in this study are available on request from the corresponding authors.
